# ^99m^Tc Labeled Glucagon-Like Peptide-1-Analogue (^99m^Tc-GLP1) Scintigraphy in the Management of Patients with Occult Insulinoma

**DOI:** 10.1371/journal.pone.0160714

**Published:** 2016-08-15

**Authors:** Anna Sowa-Staszczak, Małgorzata Trofimiuk-Müldner, Agnieszka Stefańska, Monika Tomaszuk, Monika Buziak-Bereza, Aleksandra Gilis-Januszewska, Agata Jabrocka-Hybel, Bogusław Głowa, Maciej Małecki, Tomasz Bednarczuk, Grzegorz Kamiński, Aldona Kowalska, Renata Mikołajczak, Barbara Janota, Alicja Hubalewska-Dydejczyk

**Affiliations:** 1 Department of Endocrinology, Jagiellonian University Medical College, Kraków, Poland; 2 Department of Endocrinology, University Hospital, Kraków, Poland; 3 Department of Metabolic Diseases, Jagiellonian University Medical College, Kraków, Poland; 4 Department of Endocrinology, Medical University of Warsaw, Warszawa, Poland; 5 Department of Endocrinology and Radioisotopic Therapy, Military Institute of Medicine, Warszawa, Poland; 6 Department of Endocrinology and Nuclear Medicine, Holycross Cancer Center, Kielce, Poland; 7 Radioisotope Center POLATOM, National Centre for Nuclear Research, Otwock, Poland; Mayo Clinic College of Medicine, UNITED STATES

## Abstract

**Introduction:**

The aim of this study was to assess the utility of [Lys^40^(Ahx-HYNIC-^99m^Tc/EDDA)NH_2_]-exendin-4 scintigraphy in the management of patients with hypoglycemia, particularly in the detection of occult insulinoma.

**Materials and Methods:**

Forty patients with hypoglycemia and increased/confusing results of serum insulin and C-peptide concentration and negative/inconclusive results of other imaging examinations were enrolled in the study. In all patients GLP-1 receptor imaging was performed to localise potential pancreatic lesions.

**Results:**

Positive results of GLP-1 scintigraphy were observed in 28 patients. In 18 patients postsurgical histopathological examination confirmed diagnosis of insulinoma. Two patients had contraindications to the surgery, one patient did not want to be operated. One patient, who presented with postprandial hypoglycemia, with positive result of GLP-1 imaging was not qualified for surgery and is in the observational group. Eight patients were lost for follow up, among them 6 patients with positive GLP-1 scintigraphy result. One patient with negative scintigraphy was diagnosed with malignant insulinoma. In two patients with negative scintigraphy Munchausen syndrome was diagnosed (patients were taking insulin). Other seven patients with negative results of ^99m^TcGLP-1 scintigraphy and postprandial hypoglycemia with C-peptide and insulin levels within the limits of normal ranges are in the observational group. We would like to mention that ^99m^Tc-GLP1-SPECT/CT was also performed in 3 pts with nesidioblastosis (revealing diffuse tracer uptake in two and a focal lesion in one case) and in two patients with malignant insulinoma (with the a focal uptake in the localization of a removed pancreatic headin one case and negative GLP-1 1 scintigraphy in the other patient).

**Conclusions:**

^99m^Tc-GLP1-SPECT/CT could be helpful examination in the management of patients with hypoglycemia enabling proper localization of the pancreatic lesion and effective surgical treatment. This imaging technique may eliminate the need to perform invasive procedures in case of occult insulinoma.

## Introduction

Hypoglycemia is a decreased blood glucose concentration (less than 70 mg/dl (3.9 mmol/l)) and is connected usually with specific symptoms such as tremor, anxiety, sweating, irritability, impaired vision, weakness and fatigue. Prolonged or recurrent severe hypoglycemia may lead to impaired brain function and even death [1–3]. Except for diabetic patients hypoglycemia is not a common problem. However searching for the cause of hypoglycemia and then therapy of diagnosed underlying disease is often difficult.

If there was no previous evidence of either taking drug affecting insulin secretion or any critical illnesses (i.e. hepatic, renal or cardiac failure or severe infection), the management of such patients is still challenging for clinicians as hypoglycemia might be caused by many reasons. Endogenous hyperinsulinemia could be related to insulinoma—the occurrence of neoplasm foci deriving from the pancreatic cells secreting insulin [1]. Severe non-insulinoma hyperinsulinemic hypoglycemia could be also associated with other β-cell disorders (e.g. non-insulinoma pancreatogenous hypoglycemia, post gastric bypass hypoglycemia) [4,5]. Nesidioblastosis (islet cell hyperplasia) is typically characterized by islet hyperplasia, β-cell hypertrophy, and increased β-cell mass. There are two forms of nesidioblastosis: a focal form and a diffuse form; and many cases have a defined genetic basis [6]. While management of patients with insulinoma and nesidioblastosis is different this is important to make a proper diagnosis.

Insulinoma is the most common functioning neuroendocrine tumor of the pancreas [7–9]. The laboratory tests used in case of suspicion of insulinoma are 72 hours fasting test with measurement of serum glucose, insulin and C-peptide concentrations [7–9]. Insulinoma are usually small lesions. Despite the increasing efficacy of standard imaging examinations (computed tomography (CT) or magnetic resonance imaging (MRI)) in localization of insulinoma, there are still patients for whom results of all available techniques (including also invasive methods such as endoscopic ultrasound (EUS) or selective arterial calcium stimulation test) are negative [7–11]. The experience with the use of labeled somatostatin analogues for imaging of neuroendocrine neoplasms shows the utility of nuclear medicine methods in preoperative localization of the occult tumors. However somatostatin receptor scintigraphy is positive only in about 50–60% of benign insulinomas [12]. Therefore there is a need to develop a non-invasive diagnostic procedure, which will improve the managmenet of patients with occult insulinoma.

Radiolabelled peptides are useful for specific targeting of various neoplasms and are currently being intensively investigated as a promising strategy in oncology and endocrinology [12–15]. Glucagon-like peptide-1 (GLP-1) is a short-lived hormone produced by a specific proteolytic processing of the preproglucagon molecule in intestinal L cells. The main rout of its action is binding to a specific receptor on the surface of pancreatic islet β-cells stimulating glucose-induced insulin secretion [16]. This receptor belongs to the G-protein-coupled receptors subfamily and has been examined to be in a large quantity overexpressed in benign insulinomas [17]. To the best knowledge of authors, the first studies with iodinated GLP-1 and its antagonist exendin-(9–39) devoted to GLP-1 receptor internalization were performed in the early nineties [18]. Metabolically more stable constituents of GLP-1, most of all GLP-1 receptor agonists—exendin-3 and exendin-4, have been studied not only in mouse models but also in humans. [Lys^40^(Ahx-DTPA)-NH_2_]-exendin-4 labelled with indium-111 was the first compound based on a GLP-1 analogue developed and checked in clinical conditions. ^111^In coupled to exendin-4 via DOTA complexation was examined in individuals. Both radiopharmaceuticals were able to localise human benign insulinomas which were not detected by other diagnostic methods. In addition, the GLP-1 receptor imaging gave favourable results with a complete recovery of patients after the surgical tumour excision [19–21] in a long run. The exact preoperative localization of the insulinoma limited surgical intervention only to a precise place in the pancreas and allowed sparing healthy pancreatic tissue. Patients could return to normal life without necessity of taking medication in the form of insulin substitutes. Wild and co-workers showed however that GLP-1 scintigraphy has limited potential in case of malignant insulinoma, which overexpressed mainly somatostatin receptors [22], in contrast with its benign form. However, the successful localization of GLP-1 receptor-positive lesions in the liver and a lymph node in patients with malignant form of insulinoma by [^68^Ga]Ga-DO3A-VS-Cys^40^-exendin-4 for PET imaging was also reported [23].

GLP-1 receptor imaging with ^99m^Tc-labeled exendin-4 provides an alternative strategy to those labeled with ^111^In and ^68^Ga. Owing to the physical properties of isotope and the significantly lower tumour and organ uptake of [Lys^40^(Ahx-HYNIC-^99m^Tc/EDDA)NH_2_]-exendin-4 in comparison with [Lys^40^(Ahx-DOTA-^111^In)NH_2_]-exendin-4, the estimated effective dose of [Lys^40^(Ahx-HYNIC-^99m^Tc/EDDA)NH_2_]-exendin-4 was calculated and evaluated to be over 40 times less than the one of its ^111^In-labeled congener compound. Despite lower tumor uptake connected to the significantly less efficient internalisation, scintigraphy with [Lys^40^(Ahx-HYNIC-^99m^Tc/EDDA)NH_2_]-exendin-4 was also able to detect lesions in the pancreas with high sensitivity in vivo in an animal model [24]. In 2013 our group have presented promising results of our pilot study with the use of the new compound ([Lys^40^(Ahx-HYNIC-^99m^Tc/EDDA)NH_2_]-exendin-4) [25]. It was concluded that the GLP-1 receptor tracer labelled with ^99m^Tc has probably the same high potential to find benign insulinoma foci, however, in contrast with ones labeled with ^111^In and ^68^Ga, it is still cheaper and easier to obtain, generates lower radiation burden to patients and the staff, and, what is more, it may be potentially useful for the detection with a γ-probe in an intraoperative localization of insulinoma [25].

As it was mentioned above initial results of our pilot study (considering eleven patients) were presented previously in European Journal of Nuclear Medicine and Molecular Imaging in 2013. Currently we would like to present results of the use of scintigraphy with labeled GLP-1 analogue in the group of forty patients with hypoglycemia. The aim of this study was to assess the utility of [Lys^40^(Ahx-HYNIC-^99m^Tc/EDDA)NH_2_]-exendin-4 scintigraphy in the management of patients with hypoglycemia, particularly in the detection of occult insulinomas.

## Material

### Patients

Forty patients (25 women and 15 men; mean age 65.5 ± 12.0 years, min. 16.0 years, max. 87.0 years) with symptoms of hypoglycemia and confirmed serum glucose levels concentrations below 45 mg/dl (2.5 mmol/l) with increased or confusing results of serum insulin and C-peptide concentrations and negative or inconclcusive results of previously performed imaging examination (computed tomograpahy—CT, magnetic resonance imaging—MRI, somatostatin receptor scintigraphy—SRS and endoscopic ultrasonography—EUS), were enrolled in the study. None of the patients was treated with oral antidiabetic agents. The majority of patients were selected from patients diagnosed and treated in the Department of Endocrinology of the University Hospital in Kraków. However there were also patients from four other medical centers in Poland included in the study.

The study was approved by the local Ethics Committee of Jagiellonian University in Kraków, Poland. All patients provided a written consent in accordance with an accepted documentation, after receiving detailed information about the study procedure.

## Method

### Imaging technique

Between October 2010 and April 2014 examinations with [Lys^40^(Ahx-HYNIC-^99m^Tc/EDDA)NH_2_]-exendin-4 were performed in Nuclear Medicine Unit Department of Endocrinology University Hospital in Kraków, Poland in all patients enrolled in the study. The examination was performed to localize potential insulinoma lesions in the pancreas.

Labelling procedure was developed and lyophilized kits containing [Lys^40^(Ahx-HYNIC-^99m^Tc/EDDA)NH_2_]-exendin-4 were prepared by Radioisotope Centre POLATOM, National Centre for Nuclear Research, Otwock, Poland. Preparation of [Lys^40^(Ahx-HYNIC-^99m^Tc/EDDA)NH_2_]-exendin-4 had been described previously [22,23]. The mean activity of [Lys^40^(Ahx-HYNIC-^99m^Tc/EDDA)NH_2_]-exendin-4 injected intravenously to patients was 740 MBq. Initially examinations were acquired with a dual-head, large field of view E.CAM gamma camera with low-energy high resolution (LEHR) collimators and images were evaluated with CT examination imposed by software fusion. After the installation of the hybrid device Symbia TruePoint T16 (Siemens Healthcare) in the Department all next GLP-1 receptor examinations were acquired on that system. Basic acquisition protocol for [Lys^40^(Ahx-HYNIC-^99m^Tc/EDDA)NH_2_]-exendin-4 was established on the one hand to find the optimal acquisition time with the highest tumor to non-tumor ratio (T/nT ratio) and the lowest kidney to non-tumor ratio (K/nT ratio) at the same time, and on the other hand to perform dosimetric calculations (described in details by Sowa-Staszczak and co-workers, [25]). Finally, whole-body and SPECT/CT scans with a low dose CT protocol were performed at two time points, between 3–4 h and 5–6 h after the injection of the GLP-1 analogue labelled with ^99m^Tc. Volumetric analysis was performed to assess T/nT ratio.

Every patient was carefully checked for any adverse reaction after the injection of the tracer. Blood pressure and glycemia were monitored at several time points before and after the injection of the compound in all patients. Some of the patients (including all patients with suspicion of benign insulinoma) required a glucose infusion at the time of GLP-1 receptor imaging procedure due to low blood glucose levels (below 40 mg/dl).

The obtained images were assessed by experienced nuclear medicine specialists.

Patients with positive results of GLP-1 receptor imaging were qualified to the surgical excision of the visualised lesions. The histopathological confirmation of tumor presence and the evaluation of its type were performed after surgery.

## Results

**Fig 1:**
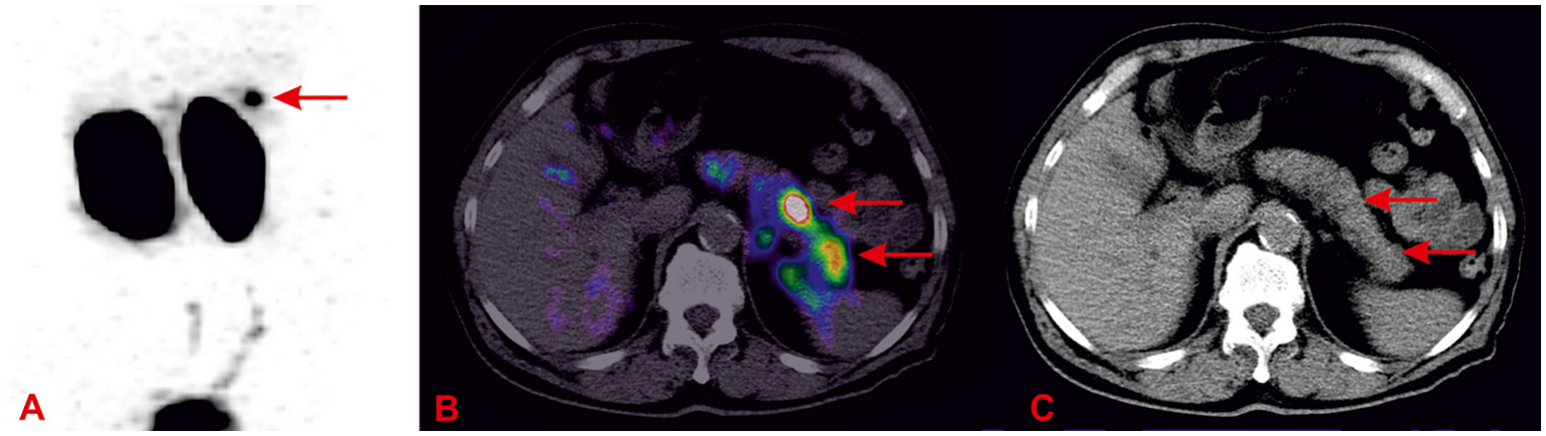
A 77-year old female—^99m^Tc-GLP1-SPECT/CT. ^99m^Tc-GLP1-SPECT/CT (Single Photo Emission Computed Tomography/Computed Tomography) revealed bifocal insulinoma (one focus is well visible on the border of the pancreatic body and tail, T/nT (Target/non-target) ratio 3.4; the second one is smaller, less visible in the pancreatic tail, T/nT ratio 1.7). Lesions were not detected by other diagnostic methods. Histopathology confirmed two foci of insulinoma: 9 mm and 2 mm in diameter. A. GLP-1 receptor imaging—MIP (*Maximum Intensity Projection*), B. Fusion of GLP-1 receptor imaging and CT—axial slice, C. CT—axial slice.

The quality of the obtained [Lys^40^(Ahx-HYNIC-^99m^Tc/EDDA)NH_2_]-exendin-4 images was evaluated as good. Positive results of GLP-1 scintigraphy were observed in 28 patients (Table 1). Among them in 18 patients postsurgical histopathological examination confirmed diagnosis of insulinoma (type G1 neuroendocrine tumours, min. 2 mm, max. 22 mm in size–Fig 1). The post-surgical resolution of symptoms was observed in all patients.

**Table 1:** Information about patients and examinations performed.

No	Initials	Sex	Age	CT/MR	GLP-1	Surgery	Final Diagnosis
**1**	J.A.	**F**	**57**	-	+	1	Insulinoma
**2**	J.W.	**M**	**16**	+/-	+	1	Insulinoma
**3**	A.P.	**M**	**75**	-	+	0	Insulinoma (not operated due to severe heart failure)
**4**	M.K.	**F**	**62**	-	+	1	Insulinoma
**5**	P.W.	**M**	**21**	+/-	+	1	Insulinoma
**6**	L.B.	**M**	**60**	-	+	0	Insulinoma (not operated—pt did not agree)
**7**	S.M.	**M**	**52**	+/-	+	0	Insulinoma (not operated; dissemination of kidney cancer)
**8**	Z.R.	**M**	**58**	-	+	1	Insulinoma
**9**	A.Z.	**F**	**44**	+/-	+	1	Insulinoma
**10**	A.L.	**F**	**39**	+/-	+	1	Insulinoma
**11**	E.K.	**F**	**35**	-	+	1	Insulinoma
**12**	E.B.	**F**	**19**	-	+	1	Insulinoma
**13**	A.B.	**F**	**31**	+/-	+	1	Insulinoma
**14**	K.B.	**M**	**77**	+/-	+	1	Insulinoma
**15**	J.A.S.	**M**	**38**	-	+	1	Insulinoma
**16**	K.J.R.	**F**	**40**	+/-	+	1	Insulinoma
**17**	K.Ł	**F**	**70**	-	+	1	Insulinoma
**18**	Z.M.	**M**	**56**	-	+	1	Insulinoma
**19**	K.C.	**F**	**16**	+/-	+	1	Insulinoma
**20**	J.S.	**M**	**57**	-	+	1	Insulinoma
**21**	B.J.	**F**	**38**	-	+	1	Insulinoma
**22**	B.O.	**F**	**40**	-	-	0	Munchausen syndrome
**23**	P.D.	**M**	**26**	-	-	0	Munchausen syndrome
**24**	R.G.	**M**	**54**	-	+	?	Lost for follow-up
**25**	B.G.	**F**	**53**	-	-	?	Lost for follow-up
**26**	K.S.	**F**	**50**	-	+	?	Lost for follow-up
**27**	T.C.	**M**	**40**	+/-	-	?	Lost for follow-up
**28**	K.F.	**F**	**50**	-	+	?	Lost for folow-up
**29**	L.M.	**F**	**60**	-	+	?	Lost for follow-up
**30**	J.B.	**F**	**64**	+/-	+	?	Lost for follow-up
**31**	G.K.	**F**	**41**	-	+	?	Lost for follow-up
**32**	J.K.	**M**	**63**	-	+	0	Not operated; Observation
**33**	K.T.	**F**	**67**	+/-	-	1	Operated; malignant insulinoma
**34**	H.K.	**F**	**47**	-	-	0	Observation
**35**	J.K.	**F**	**73**	-	-	0	Observation
**36**	K.A.	**F**	**34**	-	-	0	Observation
**37**	A.A.	**F**	**31**	-	-	0	Observation
**38**	E.C.	**F**	**69**	-	-	0	Observation
**39**	P.P.	**M**	**19**	-	-	0	Observation
**40**	R.P.	**F**	**74**	-	-	0	Observation

F female; M male; + positive result; +/- inconclusive result; CT computed tomography; MRI magnetic resonance imaging; GLP-1 scintigraphy with labeled GLP-1 analogue; 0 not operated; 1 operated;? no information

It is worth to mention, that one of the operated patients (38-year old female) was referred to the GLP-1 scintigraphy due to severe hypoglycemia after distal pancreatectomy (which was performed because of severe symptoms in spite of negative results of conventional imaging). Based on focal uptake of labelled GLP-1, patient underwent partial, selective resection of pancreatic body—histopathological examination revealed coexisting insulinoma and nesidioblastosis.

**Fig 2:**
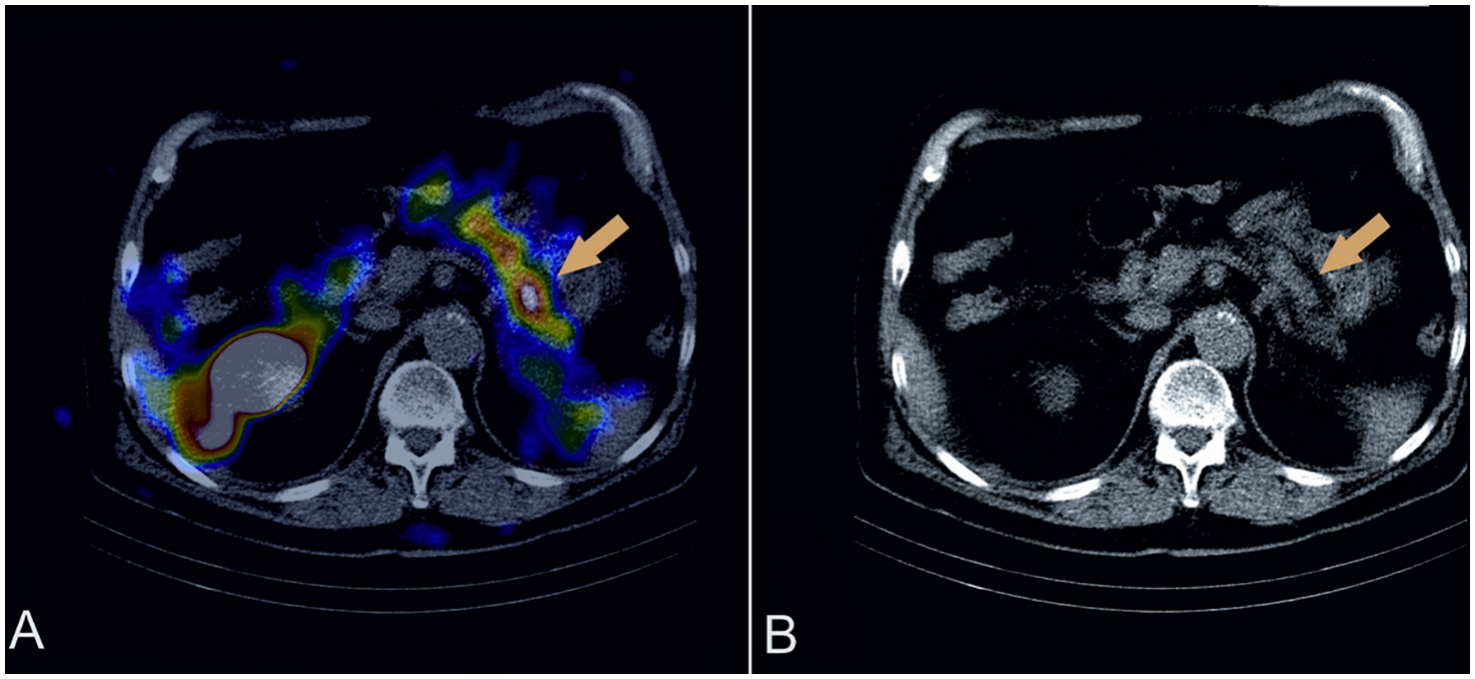
A 75-years old male with suspicion of insulinoma—^99m^Tc-GLP1scintigraphy. ^99m^Tc-GLP1scintigraphy revealed the focus of insulinoma in the pancreas—patient was disqualified from surgical excision of the tumor due to severe heart failure. The study was performed with dual-head, large field of view E.CAM gamma camera with low-energy high resolution (LEHR) collimators and images were evaluated with CT examination imposed by software fusion. A. Fusion of GLP-1 receptor imaging and CT—axial slice, B. CT—axial slice.

Three patients were disqualified from surgery. However, the positive results of GLP-1 scintigraphy, clinical and biochemical assessment indicated diagnosis of insulinoma. The first patient was disqualified from surgery due to severe heart failure (Fig 2). Hypoglycemia was controlled with diazoxide. The second patient could not have a surgical excision of pancreatic lesion due to dissemination of kidney cancer. The patient is treated with protein kinase inhibitor. The third patient did not want to be operated.

In case of one patient with positive result of the studied method description of the GLP-1 receptor examination indicated a diffuse uptake of the compound in the whole pancreas with a small focus of the slightly increased uptake in the body of the pancreas. The patient was not qualified for a surgery due to confusing results of the laboratory findings (mainly postprandial hypoglycemia with normal serum insulin and C-peptide concentrations were observed). Patient is in the observational group.

There was one patient with severe hypoglycemia, increased serum insulin and C-peptide concentrations and negative result of GLP-1 scintigraphy. The patient was qualified for surgery (lesion in the head of the pancreas was visualised in CT). The histopathological examination confirmed NET G2 tumor of the pancreas. However severe hypoglycemia was still observed after surgery. Finally, based on subsequent imaging studies, a malignant form of insulinoma was diagnosed (metastases were found in the liver).

Eight patients, among them six with positive GLP-1 scintigraphy results, were lost for follow up.

In two patients with negative scintigraphy Munchausen syndrome was diagnosed (patients were taking insulin). Other seven patients with negative results of ^99m^TcGLP-1 scintigraphy and postprandial hypoglycemia with C-peptide and insulin levels within the limits of normal ranges are in the observational group.

We would like to mention that ^99m^Tc-GLP1-SPECT/CT was also performed in three patients with nesidioblastosis (revealing diffuse tracer uptake in two and a focal lesion in one case). Two of these patients, who are currently treated with diazoxide, remain under the care of Department of Endocrinology of University Hospital in Kraków. In the third case, the GLP-1 study revealed a focal uptake of the radiopharmaceutical in the pancreas, what required further verification. The patient was qualified for a partial pancreatectomy. Unfortunately, the patient died due to surgery complications. In the analysed histopathological examination, the co-existence of nesidioblastosis and insulinoma was described.

**Fig 3:**
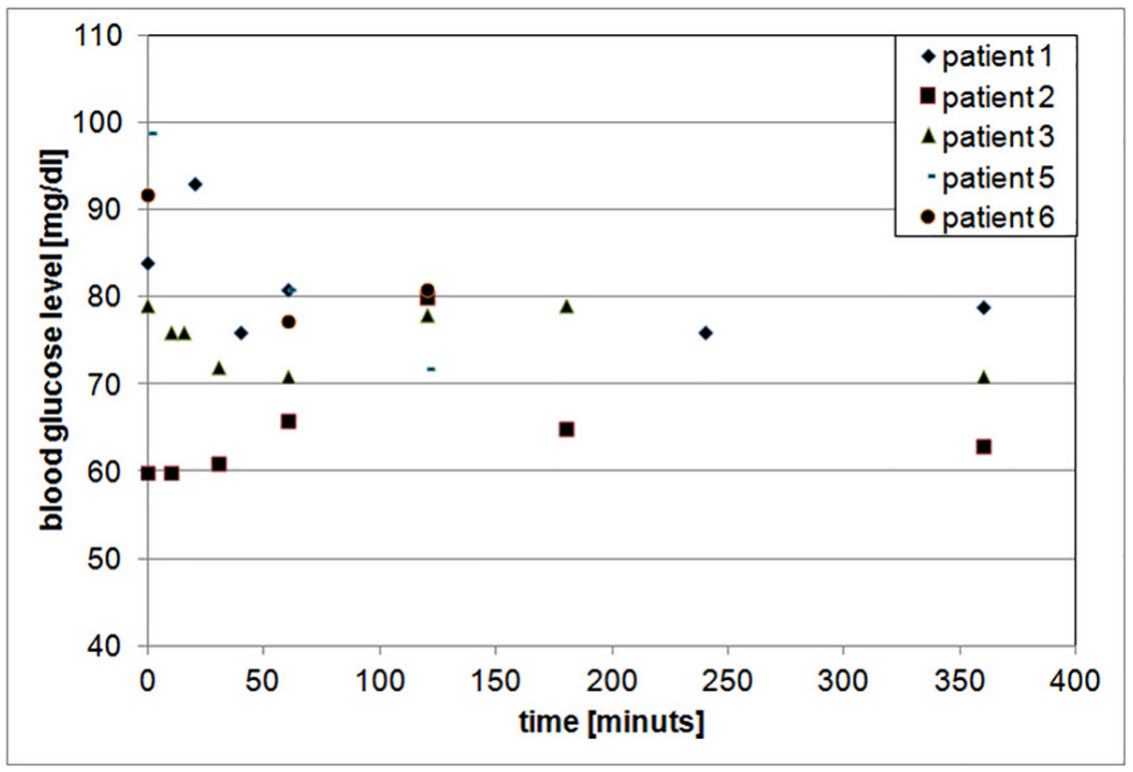
Blood glucose level versus time after injection of Lys^40^(Ahx-HYNIC-^99m^Tc/EDDA)NH_2_]-exendin-4—the group of patients with MTC.

The examination with labelled GLP-1 was also performed in two patients with malignant insulinoma. In case of the patient with suspected local recurrence of malignant insulinoma a focal accumulation of the radiopharmaceutical was revealed in the localization of removed pancreatic head (to differentiate between the recurrence or metastases to lymph nodes). There was no uptake observed in liver metastases. The patient did not want to be operated. The GLP-1 study was negative in the other patient with malignant insulinoma. No serious adverse reactions were reported by any of the patients at the time of the examination. Due to the natural disease course of insulinoma it was difficult to observe the relation between the injection of the tracer and exacerbation of hypoglycemia. Therefore, in order to assess the undisturbed impact of GLP-1 analogues on patients’ blood glucose levels, it was decided to make measurements in the group of patients with medullary thyroid carcinoma (MTC), also diagnosed by [Lys^40^(Ahx-HYNIC-^99m^Tc/EDDA)NH_2_]-exendin-4. Fig 3 presents dependence between the blood glucose level and time after injection of Lys^40^(Ahx-HYNIC-^99m^Tc/EDDA)NH_2_]-exendin-4 in the case of these patients in whom an abnormally diminished blood glucose concentration had not been observed before the examination with labelled GLP-1. The difference between the blood glucose level before the injection of Lys^40^(Ahx-HYNIC-^99m^Tc/EDDA)NH_2_]-exendin-4 and next measured values did not exceed 10% in general.

## Discussion

Preoperative localization of the insulin secreting pancreatic tumors is important for further effective surgical excision, which should be limited to the affected part of the pancreas. For the evaluation of neuroendocrine pancreatic tumors in case of computed tomography (CT) and magnetic resonance imaging (MRI) multiphase, post-contrast series should be used [26]. CT is more available to patients, but MRI seems to have superior sensitivity and specificity [26]. However, due to small size of the insulinoma tumors, standard imaging procedures such as ultrasound examination, computed tomography or magnetic resonance imaging are not always effective in the detection of tumors. For such patients with hyperinsulinemic hypoglycemia invasive techniques, such as endoscopic ultrasound (EUS) and selective arterial calcium stimulation test (SACST), may be offered [10,11]. However, these are invasive methods and they are also not always efficient in the detection of occult insulinoma [10,11].

In our paper we present results of the clinical trial with [Lys^40^(Ahx-HYNIC-^99m^Tc/EDDA)NH_2_]-exendin-4 in the detection of benign insulinoma. While the detection of insulinoma (single or rarely multifocal forms) in some cases still remains a clinical challenge for four years [Lys^40^(Ahx-HYNIC-^99m^Tc/EDDA)NH_2_]-exendin-4 (technetium-99m labelled long acting glucagon-like peptide analogue; ^99m^Tc-GLP) scintigraphy has been developed in our centre as a potential and crucial imaging technique in such cases.

As surgical excision is the only effective treatment for insulinoma patients, there is a necessity of developing alternative diagnostic strategies for cases with inconclusive results of all available imaging techniques, possibly through the use of new biomarkers. In 2008, Wild et al. [19] published the groundbreaking letter which describes the detection of two occult insulinomas using indium labelled GLP-1 analogue. Then, it was followed by successful surgery and the full recovery of the patients. In our study technetium-99m labelled GLP-1 scintigraphy proved to be also significantly helpful for patients with similar clinical situation.

In presented study GLP-1 receptor imaging with the use of Lys^40^(Ahx-HYNIC-^99m^Tc/EDDA)NH_2_]-exendin-4 was positive in case of 28 patients with hypoglycemia. We would like to emphasize that in case of 18 patients in this group results of standard imaging procedures (CT and/or MRI) were negative and in the other 10 patients results of CT and/or MRI were inconclusive. In case of 18 patients postsurgical histopathological examination confirmed diagnosis of insulinoma. Three patients with positive results of the GLP-1 receptor imaging and high probability that visualized lesions would be confirmed as insulinomas were not operated. One patient with slightly increased uptake of the tracer in the body of the pancreas was finally not qualified to the surgery and is in the observational group. Six patients with positive results of studied method were unfortunately lost for follow up. However in case of 18 patients, in whom other non-invasive imaging examinations were negative or inconclusive, it was possible to localize precisely pancreatic tumors.

Christ and Wild and co-workers [21] reported that GLP-1 receptor imaging with [Lys^40^(Ahx-DTPA-^111^In)NH2]-exendin4 detected correctly the insulinoma in 19 out of 20 patients, what results in 95% sensitivity (95% CI 75–100) and 25% specificity (95% CI 3–71). These values were also compared with results for structural imaging (CT/MRI): 47% sensitivity (05% CI 27–68) and 100% specificity (95% CI 45–100). The authors concluded that GLP-1 receptor imaging with ^111^In labelled exendin-4 is a more sensitive method for the detection of insulinoma than CT/MRI and have substantial impact on a clinical management of patients with endogenous hyperinsulinemic hypoglycemia. They also reported incidences of hypoglycemia after the injection of labelled GLP-1 analogue. Twenty (67%) out of 30 patients with suspicion of benign insulinoma required an exogenous glucose infusion to equalize the blood glucose level, but no serious episodes of hypoglycemia were observed. Similar information could be found in their previous study devoted to patients with malignant insulinoma [18].

A question, important for the safety of the patients in whom scintigrapy with labelled GLP-1 analogue is performed, has been raised whether the GLP-1 peptide, similarly to a native GLP-1, may disturb hypoglycemia counterregulation by suppression of glucagon secretion. In the study published in 2002 [27] it was shown that GLP-1 does not impair overall hypoglycemia counterregulation except for a reduction in GH responses, which is in line with other findings demonstrating pituitary actions of GLP-1. The insulinotropic action of GLP-1 was evaluated as negligible, if plasma glucose concentrations falls below 4.3 mmol/l. The author quoted also other publications supporting the statement that hypoglycemia in response to exogenous GLP-1 has only been observed under artificial conditions, i.e. the concomitant administration of GLP-1 and iv glucose. In fasting subjects or in combination with oral nutrient intake, there is no potential of GLP-1 to cause hypoglycemia [28–32]. Lerche and co-workers [33] in randomized, double-blind placebo-controlled cross-over study in eight healthy men assessed the safety, in terms of hypoglycemia, of a continuously infused pharmacological dose of native GLP-1 during long-term fasting. They concluded that the counter-regulatory response during 48 h of subcutaneous GLP-1 infusion was preserved despite long-term fasting with no apparent increased risk of hypoglycemic episodes. No reactive hypoglycemia was observed when the fast was followed by an oral glucose tolerance test. Thus use of long-acting GLP-1 analogues may not increase the risk of hypoglycaemia. However, one should remember that these experiments were conducted in healthy volunteers with normal reactions preventing hypoglycaemia.

We were trying to verify this finding in a group of patients who were not burdened by hypoglycemic episodes related to the natural course of their disease and we did not observe such phenomenon in patients with MTC without previous problems with blood glucose levels (see Fig 3).

Despite our study was dedicated mainly for localization of occult insulinoma we were trying also to assess the utility of GLP-1 receptor imaging in case of other disorders, which may cause severe or recurrent hypoglycemia. Therefore we would like to mention that the examination with radiolabelled GLP-1 could be also helpful for the differentiation of nesidioblastosis and insulinoma. Moreover, in clinical practice it should be expected that insulinoma may coexist with nesidioblastosis [25]. ^99m^Tc GLP-1 scintigraphy may be helpful in diagnosis of various forms of nesidioblastosis (considering different genetic patterns) enabling to determine the range of surgical treatment in its focal and diffuse type, if suitable. Patients with diagnosed focal form of nesidioblastosis can be completely cured by a limited pancreatectomy and the two forms of this syndrome could be distinguished by morphological criteria. Moreover, a specific genetic background of that difference was confirmed by molecular findings but genetic tests have not been still generally available [34]. It seems that the GLP-1 receptor imaging could be an alternative approach to this issue. However presented above group of patients is of course too small to draw any conclusions.

Malignant form of insulinoma is rare (up to 15% of all insulinomas), and may frequently lack GLP-1 receptors [22]. But this statement does not completely preclude usefulness of GLP-1 receptor imaging in the case of malignant insulinoma, as it was shown by Eriksson and co-workers [23]. In our Center the GLP-1 receptor imaging with the use of Lys^40^(Ahx-HYNIC-^99m^Tc/EDDA)NH_2_]-exendin-4 was performed in 3 patients with malignant insulinoma. Two other patients were diagnosed with malignant insulinoma before the examination with labeled GLP-1 analogue. One of those patient was included in the study—the metastatic lesions in the liver occurred after excision of the pancreatic tumor. The results of the [Lys^40^(Ahx-HYNIC-^99m^Tc/EDDA)NH_2_]-exendin-4 scans were very interesting: for two patients the scan was negative for both primary tumor and liver metastases, in the next case the focal accumulation of the tracer was revealed in the localization of a removed pancreatic head (recurrence of the disease) but metastases were negative. This phenomenon inidcates the different biology of primary lesions and metastases, already well known in neuroendocrine neoplasms.

At the end it is worth to emphasize a high tumor to non-tumor (T/nT) ratio for Lys^40^(Ahx-DTPA-^111^In)NH2]-exendin-4, which we observed in our study. For optimizing the acquisition protocol, amino acids, Gelofusine, fragmented albumin or other substances should be used for blocking tracer uptake by proximal kidney tubules, because of a very high kidney to non-tumor ratio. It will probably improve diagnostic efficiency of the GLP-1 scintigraphy, especially when small pathological lesions are located in proximity to the kidneys or in the pancreatic head and tail [18,25]. It should be emphasized that only the use of hybrid technology SPECT/CT allows proper lesions assessment in the scintigraphic scans. The best quality images and the most suitable relation of T/nT ratio to K/nT were obtained 5–6 hours after [Lys^40^(Ahx-HYNIC-^99m^Tc/EDDA)NH_2_]-exendin-4 (740MBq) injection. Additional delayed images could be still needed in patients with negative early scans. In previous paper [25], on the basis of biokinetics and dose assessment studies, it was reported that the average effective dose for patients after GLP-1 analogues labelled with ^99m^Tc is comparable with the radiation dose to patients after SRS performed with ^99m^Tc-EDDA/HYNIC-Tyr^3^-octreotide. Despite some technical difficulties to overcome (a very high kidneys’ uptake), the new compound seems to be an effective new tracer for clinical practice and appears to be safe for the patient and the staff.

Due to a very high density of GLP-1 receptors in benign insulinomas, it is possible that examinations with all GLP-1 tracers (different GLP-1 analogues labelled with ^99m^Tc, ^111^In, ^68^Ga, ^18^F or ^64^Cu) will be characterized by a similar, very high diagnostic accuracy. As ^18^F-DOPA PET has been also presented as the preferred compound for insulinoma detection [35], the comparative studies are needed to prove diagnostic utility of both methods.

## Conclusions

In our study ^99m^Tc-GLP1-SPECT/CT proved to be helpful imaging method in the management of patients with severe hypoglycemia. ^99m^Tc-GLP1-SPECT/CT could be considered in the former stages of diagnostic schemes to optimize the procedures and to enable, by proper localization of the pancreatic lesion, effective, healthy pancreatic tissues sparing, surgical treatment. In case of occult insulinomas this imaging technique may eliminate also the need to perform invasive procedures, which also in some cases have limited efficacy.
